# Quantifying effectiveness and best practices for bumblebee identification from photographs

**DOI:** 10.1038/s41598-023-41548-w

**Published:** 2024-01-10

**Authors:** A. M. Colgan, R. G. Hatfield, A. Dolan, W. Velman, R. E. Newton, T. A. Graves

**Affiliations:** 1 U.S. Geological Survey, Northern Rocky Mountain Science Center, 38 Mather Drive, West Glacier, MT 59936 USA; 2 Xerces Society for Invertebrate Conservation, 628 NE Broadway, Suite 200, Portland, OR 97221 USA; 3 Boise, ID 83702 USA; 4 Bureau of Land Management, 5001 Southgate Drive, Billings, MT 59101 USA

**Keywords:** Community ecology, Conservation biology

## Abstract

Understanding pollinator networks requires species level data on pollinators. New photographic approaches to identification provide avenues to data collection that reduce impacts on declining bumblebee species, but limited research has addressed their accuracy. Using blind identification of 1418 photographed bees, of which 561 had paired specimens, we assessed identification and agreement across 20 bumblebee species netted in Montana, North Dakota, and South Dakota by people with minimal training. An expert identified 92.4% of bees from photographs, whereas 98.2% of bees were identified from specimens. Photograph identifiability decreased for bees that were wet or matted; bees without clear pictures of the abdomen, side of thorax, or top of thorax; bees photographed with a tablet, and for species with more color morphs. Across paired specimens, the identification matched for 95.1% of bees. When combined with a second opinion of specimens without matching identifications, data suggested a similar misidentification rate (2.7% for photographs and 2.5% specimens). We suggest approaches to maximize accuracy, including development of rulesets for collection of a subset of specimens based on difficulty of identification and to address cryptic variation, and focused training on identification that highlights detection of species of concern and species frequently confused in a study area.

## Introduction

Bumblebees can be difficult to identify. For example, novice citizen scientists may misidentify up to 50% of bumblebees^1^. Both species misidentification and lack of detection of a species can create challenging, persistent problems for assessment of pollinator species richness, distributions, habitat relationships, and community dynamics^2,3^. We need species-level data to understand how changes in pollinator presence, abundance, and community structure affect pollination. Species-level pollinator data enable researchers to identify a link between a long-term decline in the species richness of pollinator communities and a decline in the quantity (number of pollinator visits) and quality (fidelity of pollinators) of pollination^4^. With their large, fuzzy bodies, bumblebees are some of the most effective pollinators, especially in colder climates^5,6^. Many bumblebee species are in decline^7,8^, and declining bumblebee populations could have variable consequences for pollination, depending on the species. Implications of species-level declines for humans and ecosystems make accurate species-level bumblebee data especially valuable^9^.

Effective bumblebee conservation and management depend on good species-level identifications. The primary conservation legislation in both the United States (the U.S. Endangered Species Act) and Canada (the federal Species at Risk Act) focus on species as their primary actionable taxonomic unit^10,11^. Listing decisions under the Endangered Species Act depend on species-specific data on the ecology, current conditions, and predicted conditions^12^. Beyond legislative requirements, species-specific data can be used to tailor conservation strategies to the needs and vulnerabilities of threatened species. Different species, even within a single genus, can have divergent responses to stressors and change. For example, a study that looked at changes in the abundances and ranges of eight North American *Bombus* species across their ranges found declines in *B. occidentalis* Greene*, B. pensylvanicus* (DeGeer)*, B. affinis* (Cresson)*,* and *B. terricola* (Kirby) but stability in *B. bifarius* (Cresson), *B. vosnesenskii* (Radoszkowski)*, B. impatiens* (Cresson)*,* and *B. bimaculatus* (Cresson)^13^. Targeted, efficient, and effective conservation actions rely on identifying population trends of individual species and the factors driving those trends^14,15^. For bees in the western U.S., many areas remain under-surveyed^8,16^. Multiple initiatives are working to fill these data gaps. However, the specialized expertise required to identify bumblebee species remains a limiting factor in meeting data needs^17–19^.

Many bumblebee species are morphologically similar and identifying species can be difficult in the field^20,21^. Traditionally, bee research has involved the collection of specimens, which are pinned and preserved. Although collecting specimens can separate the time-intensive field sampling step from the identification step that requires expertise, a reliance on specimens has several disadvantages^22^. Specimen collection requires destructive sampling, which in the case of the endangered rusty patch bumblebee (*B. affinis* (Cresson)) requires a specialized permit^23^ and could potentially further imperil a species the research program is intended to protect. Although one study found no decrease in bee abundance, species richness, or evenness as a result of destructive sampling, this study occurred in an area with abundant floral resources^24^. Multiple bumblebee species are under consideration for listing, and many people have greater concerns about the impacts of sampling declining species as a recent review of lethal sampling discussed^25^. This is especially true in populations on the edge of the range, in marginal habitat, or that may have other stressors^26^. Likewise, logistical challenges with collecting limit the people and locations to experts and museums with resources to carefully collect, process, pin, and database specimens to ensure the specimens are identifiable and the data are usable^19,25^.

In recent years, driven partly by the need to increase baseline understanding of pollinator distributions, taking photographs has emerged as an alternative to collecting specimens. Documenting bumblebees with photographs engages citizen scientists, creates excitement for bees, educates the public on bee diversity, fills holes in bumblebee distribution data, and informs local and broader conservation. For example, several bumblebee atlas projects seek to understand distributions of multiple species using a solid design with strong inference by engaging trained volunteers to conduct surveys following a standardized protocol^27^. Volunteers upload photographs of bumblebees detected during surveys to bumblebeewatch.org, where experts verify identifications. If bumblebees are not detected during surveys, lack of detection is also recorded. Atlas efforts are ongoing in 15 states and at least five more will begin in 2024^27^. Data from the program have been used to inform petitions for listings, document changes in species abundances and range extents^28^, and identify potential causes of species declines^29^. Similarly, researchers are using platforms like iNaturalist to engage naturalists and community scientists in observing and documenting many different taxa. By involving more people, citizen science efforts can increase the chance that rare and threatened species will be detected, and likewise also document where these species are not detected, despite standardized effort^30^. In one case, photos uploaded to iNaturalist led to the first documented observation of a very rare bumblebee species (*B. irisanensis* (Cockerell)) since the 1990s^31^. Increases in observations may also improve distribution models^8^.

The bounty of observations generated by citizen science projects has benefited conservation science but has also raised questions about the quality of the data these projects generate. The potential for misidentification of species concerns managers and scientists alike because misidentifications can have serious repercussions^32^. False positive misidentifications of Eurasian lynx in the Alps and wolves in northern Montana likely led to overestimations of the species range^33,34^. Such errors have implications for management actions and the survival of threatened species^12^.

Existing literature about photo identification of bumblebees has mostly focused on the ability of citizen scientists to identify bumblebee species. These studies generally measure the accuracy or consistency of the citizen scientists’ identifications against an expert identification^35^. Such methods assume the expert identifications are accurate. Although limited research exists on misidentification rates among experts, this may be a faulty assumption^36^. Given the widespread reliance on expert verifications of photo-based bumblebee identifications, the potential implications of misidentifications, and the challenges of identifying bumblebee species, we urgently need to evaluate the efficacy of expert identifications of bumblebees from photographs and understand what situations may be better suited to alternate approaches.

We know of no studies that have directly compared photo identifications with specimen identifications of bumblebees. A 2019 study from the UK compared identifications made by citizen scientists with verifications by experts, but the expert verifications were based solely on photos and no attempt was made to evaluate the expert identifications^1^. Another study found that experts were able to identify 88% of photographed bees in their study in Germany to the species level but did not compare accuracy of identifications^37^.

Here, we evaluate the strengths and limitations of expert identifications of *Bombus* species from field photographs recorded in Montana, North Dakota, and South Dakota. Using a collection of bumblebees with independent identification of paired photographs and specimens and additional bumblebees with only photographs sampled, we: (1) evaluate how frequently species can be determined from photographs and specimens (identifiability) and how frequently determinations from photographs agreed with those made from specimens (agreement); (2) present species-specific data for 20 species, including 5 species of concern; (3) identify factors that affect the identifiability and agreement scores of photographs; and (4) provide suggestions for best practices when using photographs for identifications based on our results.

## Results

### Efficacy

**Figure 1:**
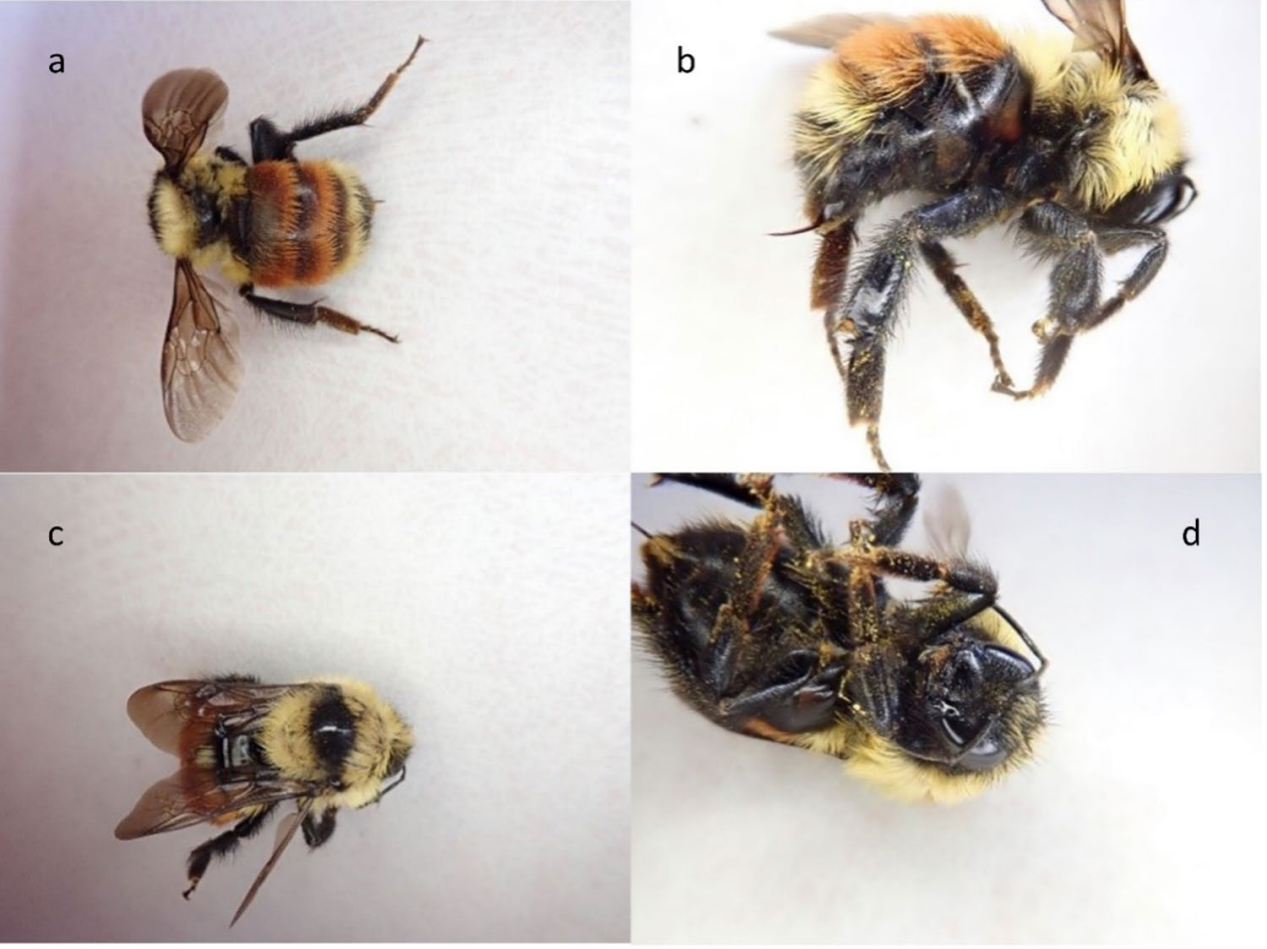
Example of high-quality photographs (USGS). In this set of four photos of a female *B. rufocinctus* all eight key diagnostic features are clearly visible in at least one of the photographs: the color of the segments on the abdomen and the posterior two segments of the abdomen (**a** and **b**), the color of the abdomen under the wings (seen from above) (**a**), the color of the thorax between the wings (**c**), the side of the thorax (**b**), the color of the face (**d**), the color of the top of the head (**c**), and the hind leg (**b**). Note “color” refers to the color of the hairlike setae on the respective section of the body.

We measured the efficacy of bumblebee identification from field photographs in two ways: whether a species could be identified (“identifiability”) and whether the species identification from a specimen matched the independent species identification from photographs of that bee (“agreement”). Bumblebees were identified to species based on four to six photographs per bee for 1310 of 1418 bumblebees (identifiability = 92.4%). Figure 1 has example photographs. In comparison, specimens were identified to species for 551 out of the 561 specimens (identifiability = 98.2%). We had paired specimens and photographs for 561 bumblebees. Six of these bees were identified from photographs but not from specimens, whereas 65 bees were identified from specimens but not from photographs. Four bees were not identified from either specimens or photographs. This left us with 486 bees with independent identifications from both a specimen and photographs. Among these bees, the species identification from photographs agreed with the identification from the specimen for 462 of 486 bees (agreement = 95.1%).

### Efficacy by species and sex

We calculated species- and sex-specific identifiability and agreement scores for the 545 bumblebees with specimen identifications and sex identifications. Our dataset included 20 species based on specimen identifications (Table 1). We report species- and sex-specific scores using species groups defined by the specimen identifications, but photo identifications included two additional species (*B. frigidus* Smith and *B. sitkensis* Nylander, Table 1). Sample sizes varied widely across species and sex. We had at least ten males and ten females for five species (*B. fervidus* (Fabricius)*, B. griseocollis* (DeGeer)*, B. huntii* (Greene)*, B. insularis* (Smith)*,* and *B. nevadensis* (Cresson). We had at least ten female individuals for four other species (*B. bifarius, B. centralis* (Cresson), *B. flavifrons* (Cresson)*,* and *B. rufocinctus* (Cresson). The remaining species and sexes accounted for only 74 of the 545 bumblebees (13.6%). For many of these species and sexes, we only had one or two bees, leading to limited inference and wider ranges for both identifiability (0–100%) and agreement (50–100; Table 1).

**Table 1:** Efficacy of photo identifications by species and sex for 551 bees collected 2019–2021 in Montana, North Dakota, or South Dakota, USA, based on percent of bees identified (% Identified) to species from photographs.

Specimen ID	% Identified female (*n*)	% Identified male (*n*)	% Agreement female (*n*)	Females confused with	% Agreement male (*n*)	Males confused with
*B. appositus*	75 (4)	100 (1)	100 (3)		100 (1)	
*B. bifarius*	92.2 (51)	100 (3)	100 (47)		100 (3)	
*B. bimaculatus*	100 (1)	0 (1)	100 (1)			
*B. centralis*	84.6 (13)	57.1 (7)	90.9 (11)	*B. huntii*	100 (4)	
*B. fervidus*	95.5 (44)	95.7 (23)	100 (42)		100 (22)	
*B. flavidus*	100 (3)	83.3 (6)	66.7 (3)	*B. insularis*	100 (5)	
*B. flavifrons*	75 (20)	100 (1)	73.3 (15)	*B. appositus, B. centralis*	100 (1)	
*B. fraternus*	100 (1)		100 (1)			
*B. griseocollis*	98 (50)	95.2 (42)	100 (49)		97.5 (40)	*B. rufocinctus*
*B. huntii*	82.5 (57)	80 (20)	95.7 (47)	*B. rufocinctus, B. sylvicola*	81.2 (16)	*B. bifarius, B. rufocinctus, B. sylvicola*
*B. impatiens*	100 (1)		100 (1)			
*B. insularis*	94.1 (17)	65.4 (26)	100 (16)		100 (17)	
*B. melanopygus*	100 (3)		100 (3)			
*B. mixtus*	77.8 (9)	100 (1)	85.7 (7)	*B. rufocinctus*	0 (1)	*B. sitkensis*
*B. nevadensis*	100 (31)	80.6 (31)	96.8 (31)	*B. pensylvanicus*	100 (25)	
*B. occidentalis*	50 (2)		100 (1)			
*B. pensylvanicus*	100 (7)		85.7 (7)	*B. fervidus*		
*B. rufocinctus*	91.3 (46)	75 (8)	85.7 (42)	*B. frigidus, B. griseocollis, B. huntii, B. sylvicola*	100 (6)	
*B. sylvicola*	100 (5)	100 (1)	60 (5)	*B. rufocinctus*	100 (1)	
*B. vagans*	100 (9)	100 (1)	100 (9)		100 (1)	

Percent agreement is the percent of bees for which the photo and specimen identifications matched. If the photo identification did not match the specimen identification, we report the photo identification in the “confused with” column. We excluded five bees for which sex was unknown.

**Figure 2:**
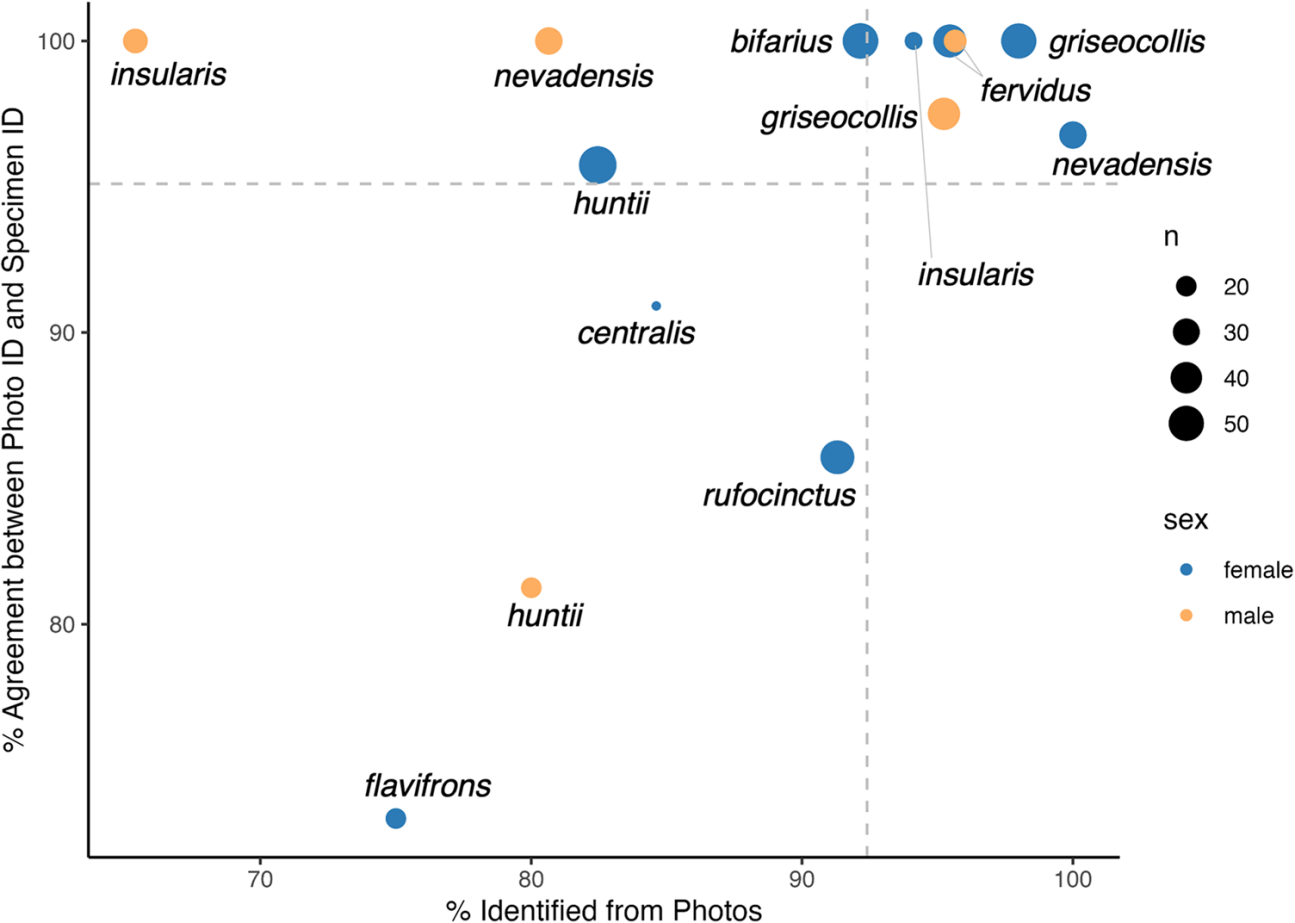
Efficacy of photo identifications for each species and sex with at least ten bees based on identifiability (x axis) and agreement (y axis). The vertical dashed line is the average percent identified and the horizontal is the average percent agreement. See Table 1 for full results.

Among species and sexes for which we had at least ten individuals, six had better than average identifiability and agreement. These were: *B. griseocollis* males and females, *B. fervidus* males and females, *B. nevadensis* females, and *B. insularis* females (Fig. 2). We found *B. bifarius* females, *B. huntii* females, *B. nevadensis* males, and *B. insularis* males were less likely to be identified but still had above average agreement when they were identified. Finally, we found below average identifiability and agreement for *B. centralis* females, *B. rufocinctus* females, B*. huntii* males, and B. *flavifrons* females.

Overall, we were able to identify female bees from photographs more frequently than male bees (94.6% of the 1000 females versus 89.7% of the 397 males). Sex was not determined for 21 individuals. For identified male bees, photographic identifications were slightly more likely to agree with specimen identifications (96.5% for the males versus 94.4% for the females of the specimens with sex determinations).

**Figure 3:**
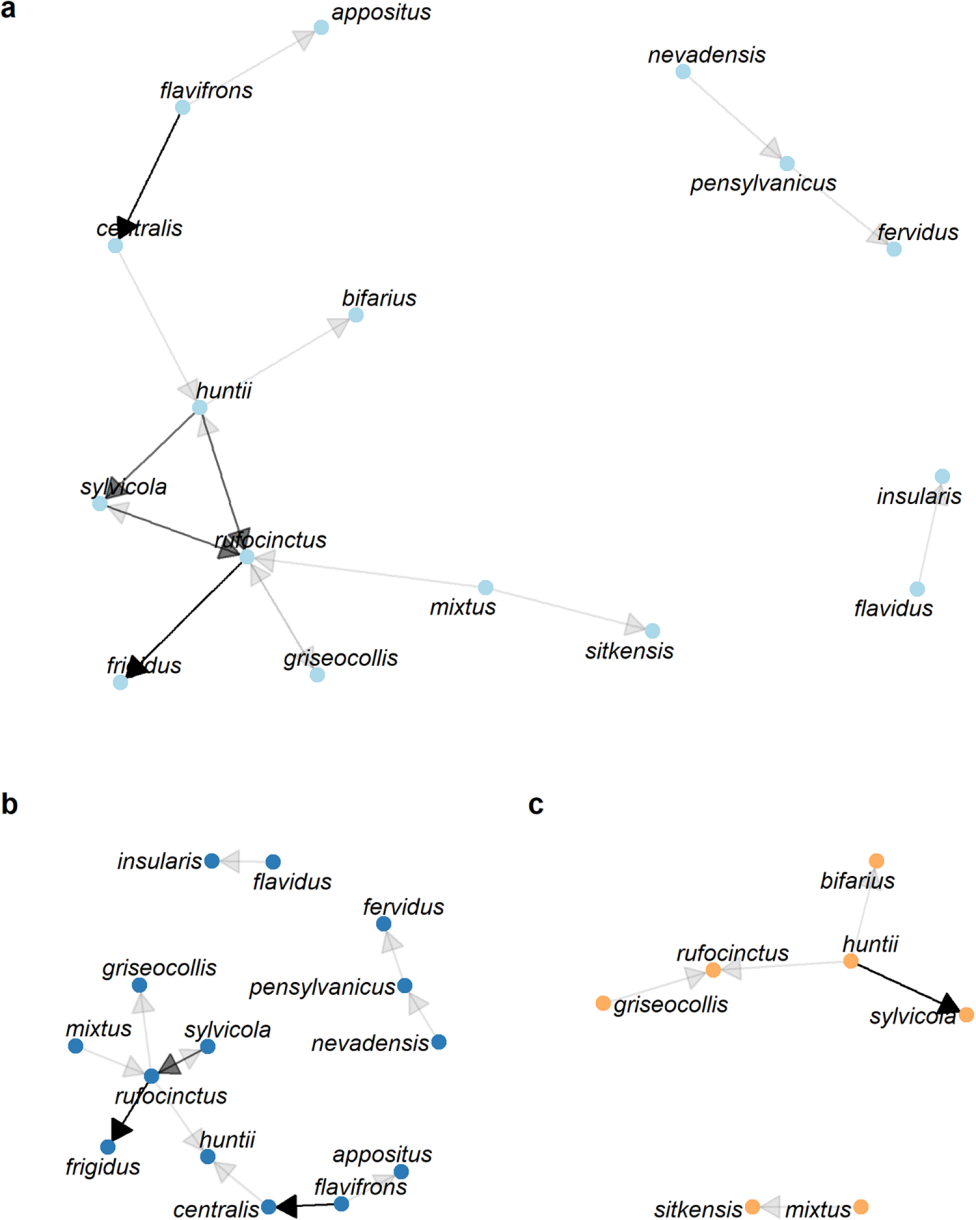
Network diagram showing species confusions for (**a**) all bumblebees, (**b**) female bumblebees, (**c**) male bumblebees. Arrows point from the specimen identification to the photo identification. Darker arrows indicate more frequent confusion between two species.

Ten species of bumblebees had species identification from photographs that differed from the identification of the specimen. Of these, four species (*B. rufocinctus* females, *B. flavifrons* females, *B. huntii* males and females, and *B. mixtus* (Cresson) males and females) were identified via photo as more than one other species (Fig. 3). For example, specimens identified as *B. rufocinctus* were identified from photographs as *B. frigidus, B. griseocollis, B. huntii,* and *B. sylvicola* (Kirby). Similarly, *B. huntii* specimens were identified from photographs as *B. bifarius*, *B. rufocinctus*, and *B. sylvicola,* and *B. flavifrons* specimens were identified from photographs as *B. appositus* (Cresson), and *B. centralis*. Notably, of the 24 instances where photograph and specimen identifications disagreed, 12 (50%) involved *B. rufocinctus* (Supplement Table 1).

To better understand the causes of disagreements between identifications from specimens and identifications from photographs, we sent a subset of specimens to another expert for a second opinion. This subset included 23 of the 24 bumblebees for which the identification based on the specimen did not match the identification based on the photograph. The original specimen identification was on the specimen label. For these 23 bees, the second specimen identification agreed with the original specimen identification in 11 instances (47.8%) and with the original photo identification in ten instances (43.4%). In the remaining two instances (8.7%), the expert identified the specimen as a third species that did not match either original identification. Using these second specimen identifications, the initial specimen identification, and the photo identification, we determined a “consensus identification” for each bee defined as the species identification supported by at least two experts. Photo-based identifications were part of the consensus identification 97.3% of the time whereas the initial specimen-based identifications were part of the consensus identification 97.5% of the time (Supplement Fig. 1). In three cases no consensus identification was available because either all three experts had different identifications (n = 2) or the two initial identifications disagreed and no second opinion was available (n = 1). Overall, this suggests an initial misidentification rate of ~ 2.5% for both photographs and specimens.

### Species of concern

Our paired specimen and photograph dataset included four species listed by the International Union for Conservation of Nature (IUCN) as vulnerable or endangered (Supplement Table 3): *Bombus fervidus, B. fraternus* (Smith)*, B. pensylvanicus, and B. occidentalis*^38^. Except for *B. fervidus* (*n* = 68) these species were represented by small sample sizes (*B. fraternus*: *n* = 1, *B. occidentalis*: *n* = 2, and *B. pensylvanicus*: *n* = 7). *Bombus fervidus* had an identifiability score of 94.1% and agreement score of 100%. Our single *B. fraternus* specimen was successfully identified from both specimen and photographs. Of two specimens identified as *B. occidentalis,* one was successfully identified from photographs, and one was not. In addition, a third bee that did not have an initial specimen identification was identified using photographs as *B. occidentalis*, which matched the second opinion identification of the specimen. Finally, of the seven specimens identified as *B. pensylvanicus*, six photo identifications agreed but one was identified as *B. fervidus.* Our data also included one bee consistently identified as *B. impatiens* from both the specimen and photographs. Concern exists that *B. impatiens,* native to eastern North America, may become invasive in western North America if it escapes and expands from current commercial use in hothouses.

### Characteristics influencing identification

**Figure 4:**
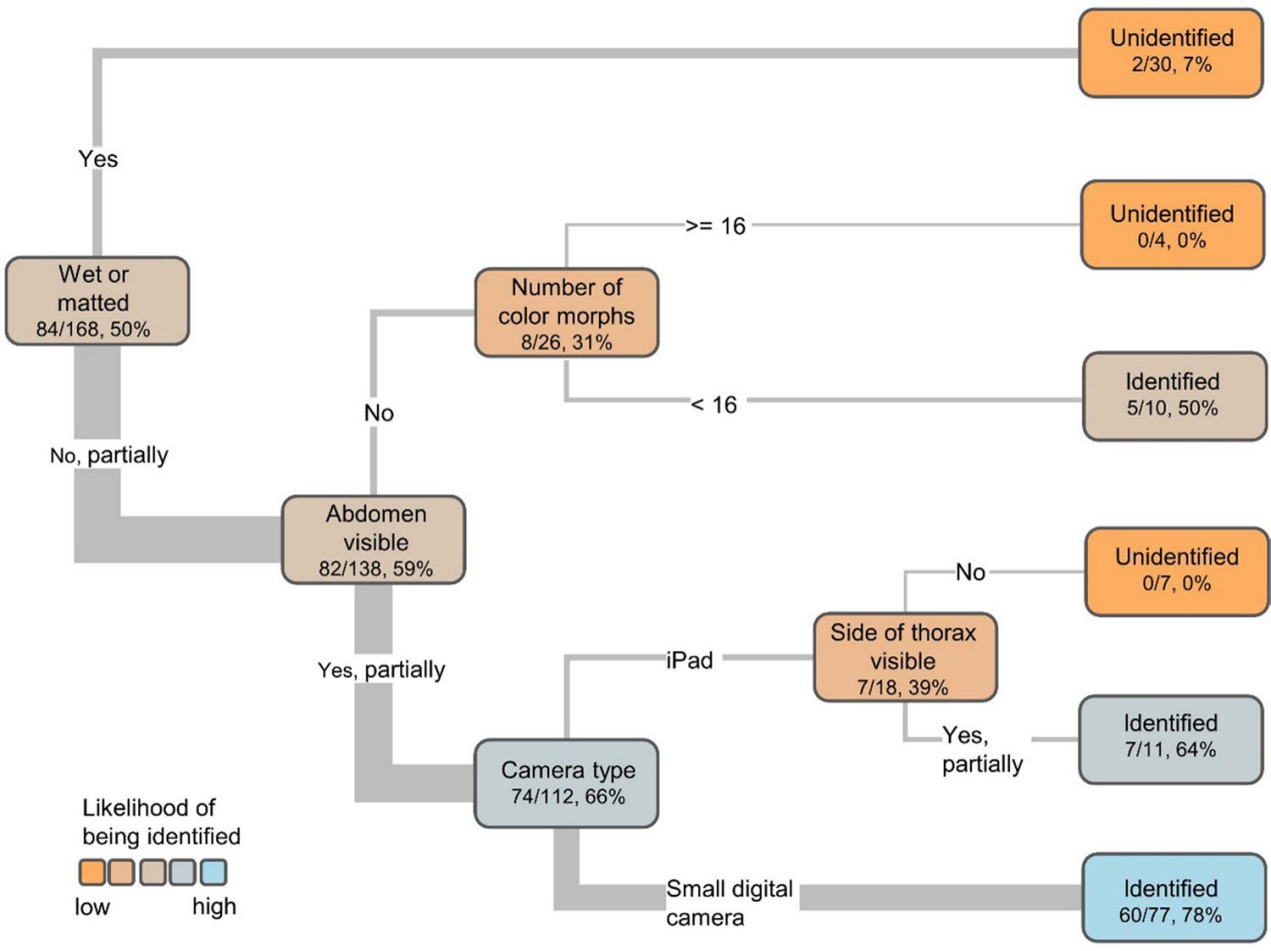
Results of classification tree analysis showing characteristics influencing whether bumblebees were identified from photographs. Analysis was run on a representative subset of data with even numbers of identified (n = 84) and unidentified (n = 84) bees and balanced representation of species and sexes in the identified and unidentified groups. Numbers represent the number of identified bees, the total number of bees, and the percent identified for each group.

A classification tree analysis showed five characteristics influencing whether experts identified a species: (1) whether a bee was wet and matted; (2) whether the abdomen was clearly visible; (3) whether the photographs were taken with a small digital camera or an iPad; (4) the number of color morphs for the species; and (5) whether the side of the thorax was clearly visible (Fig. 4). We ran the classification tree on a representative subset of the data with balanced numbers of identified and unidentified bees (n_identified_ = 84, n_unidentified_ = 84, n_total_ = 168; supplementary Table 4). The pruned classification tree had a depth of four, included five splits, and explained 76% of the variance in the identifiability data (Residual error = 0.333, CV Error = 0.655, SE = 0.0724, Misclass rate = 0.167, CV = 0.327, *n* = 168).

**Figure 5:**
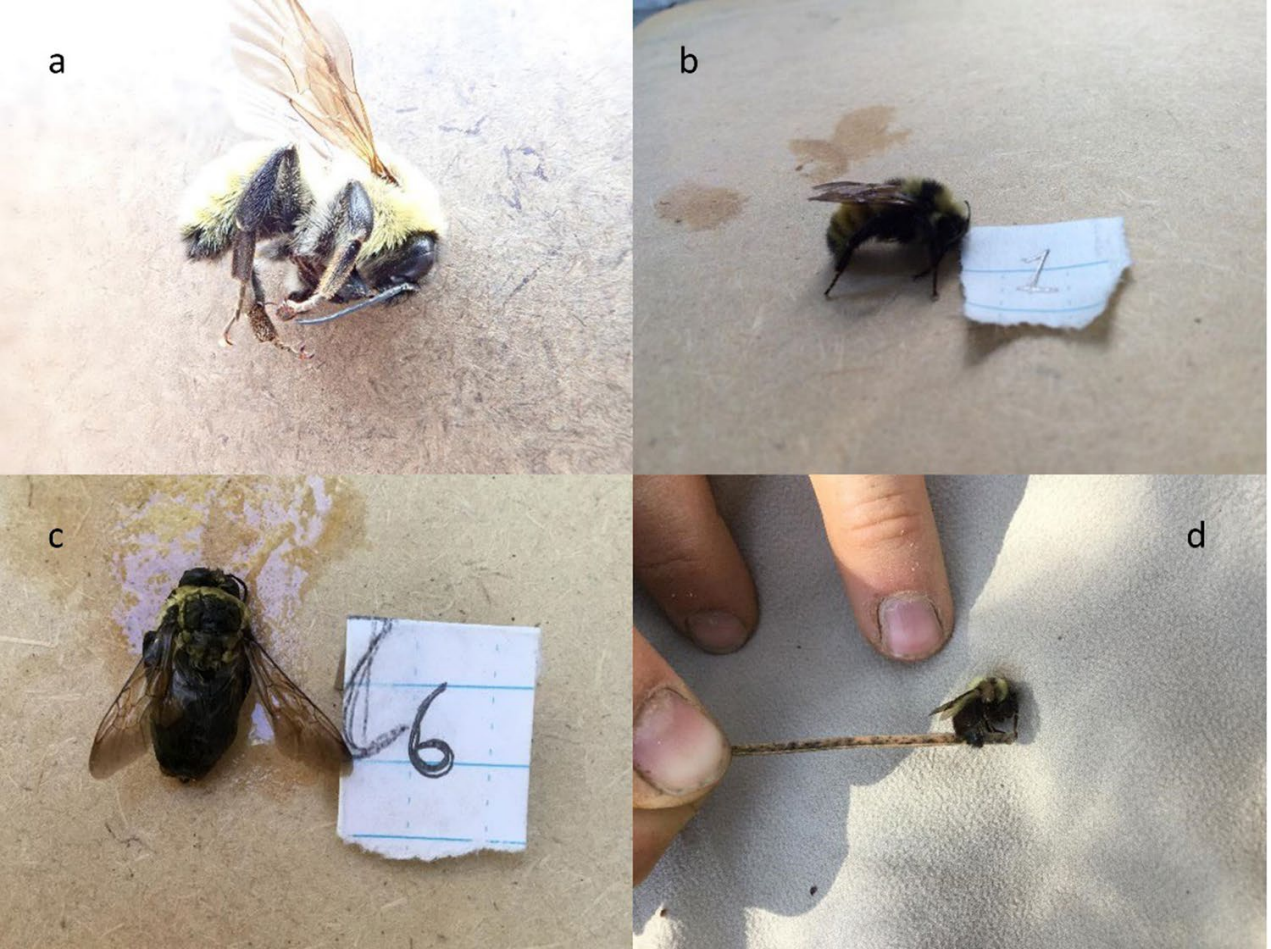
Examples of low-quality photographs (USGS). The most common causes of low-quality in bumblebee photographs included (**a**) overexposure, (**b**) underexposure and or blurry photos, (**c**) wet bees, and (**d**) zoomed out photos with insufficient resolution.

Whether a bee was wet and matted in the photographs explained 31% of the variance in the identifiability data. Only two out of 30 bees (7%) that were wet or matted were identified, whereas 82 of the 138 bees (59%) that were not or only partially wet and matted were identified. The second splitting criteria was whether the colors of the setae on the bumblebee’s abdomen were clearly visible in at least one photograph. Setae are hairlike structures made of chitin; to minimize jargon we will refer to setae as ‘hairs’ hereafter. Photos without clearly visible colors of the ‘hairs’ on the abdomen included photos with poor exposure or lighting and photos with low resolution, either due to the camera or because the photographer did not fill the photo frame with the bee (Fig. 5). Only eight out of 26 bees (31%) for which the colors of the abdominal ‘hairs’ were not clearly visible were identified, whereas 74 out of 112 (66%) of the bees with visible abdominal ‘hair’ colors were identified. For bees with unclear color of the abdominal ‘hairs,’ the next split was the number of color morphs recorded for bees of the species. Bees without a good abdomen photograph were more likely to be identified (5 of 10, 50%) if they had fewer color morphs; no bees were identified (0 of 4) otherwise. In cases where the abdominal ‘hair’ color was visible, bees photographed with a small digital camera such as the Olympus TG-5 were more likely to be identified (60 out of 77, 78%) than those photographed using an iPad (7 out of 18, 39%). Of those photos taken with an iPad, bees with a clear photograph of the ‘hairs’ on the side thorax were more likely to be identified (7 out of 11, 64%) than those with no clear photograph of this area (0 out of 7, 0%). Alternate splits and improvement scores are reported in Table 2.

**Table 2:** Alternative splits of classification trees and improvement scores for each criterion.

	Best split		First alternative		Second alternative		Third alternative		Fourth alternative	
	Criterion	I	Criteria	I	Criteria	I	Criteria	I	Criteria	I
Node 1	Wet matted < 0.75	13.72	Abdomen < 0.25	12.76	Thorax < 0.25	10.50	Hind leg < 0.25	9.05	Face color < 0.25	8.71
Node 2	Abdomen < 0.25	5.26	Camera category	4.49	Head color < 0.25	3.83	Thorax < 0.25	3.54	Hind leg < 0.25	3.41
Node 4	Camera category	4.45	Head color < 0.25	2.77	Hind leg < 0.75	1.62	Thorax < 0.25	1.58	Abdomen < 0.75	1.47
Node 5	n color morphs < 15.5	1.43	Face color < 0.25	1.33	Hind leg < 0.25	0.87	Head color < 0.25	0.85	Striped bee < 0.25	0.81
Node 9	Thorax side < 0.25	3.46	Face color < 0.25	1.34	Cuckoo	1.30	n color morphs < 8.5	1.05	Thorax < 0.75	0.76

I = Improvement Score, which is a relative measure of how much each split improves the homogeneity of the sub nodes. For instance, at node 1, splitting based on “wet and matted” improved the homogeneity of the resulting sub nodes by an index of 13.72 whereas splitting by “abdomen” improved the homogeneity by slightly less (12.76). Photo quality characteristics including “wet matted” and the visibility of key features like the abdomen were ocularly scored between 0 and 1 (clearly visible).

**Figure 6:**
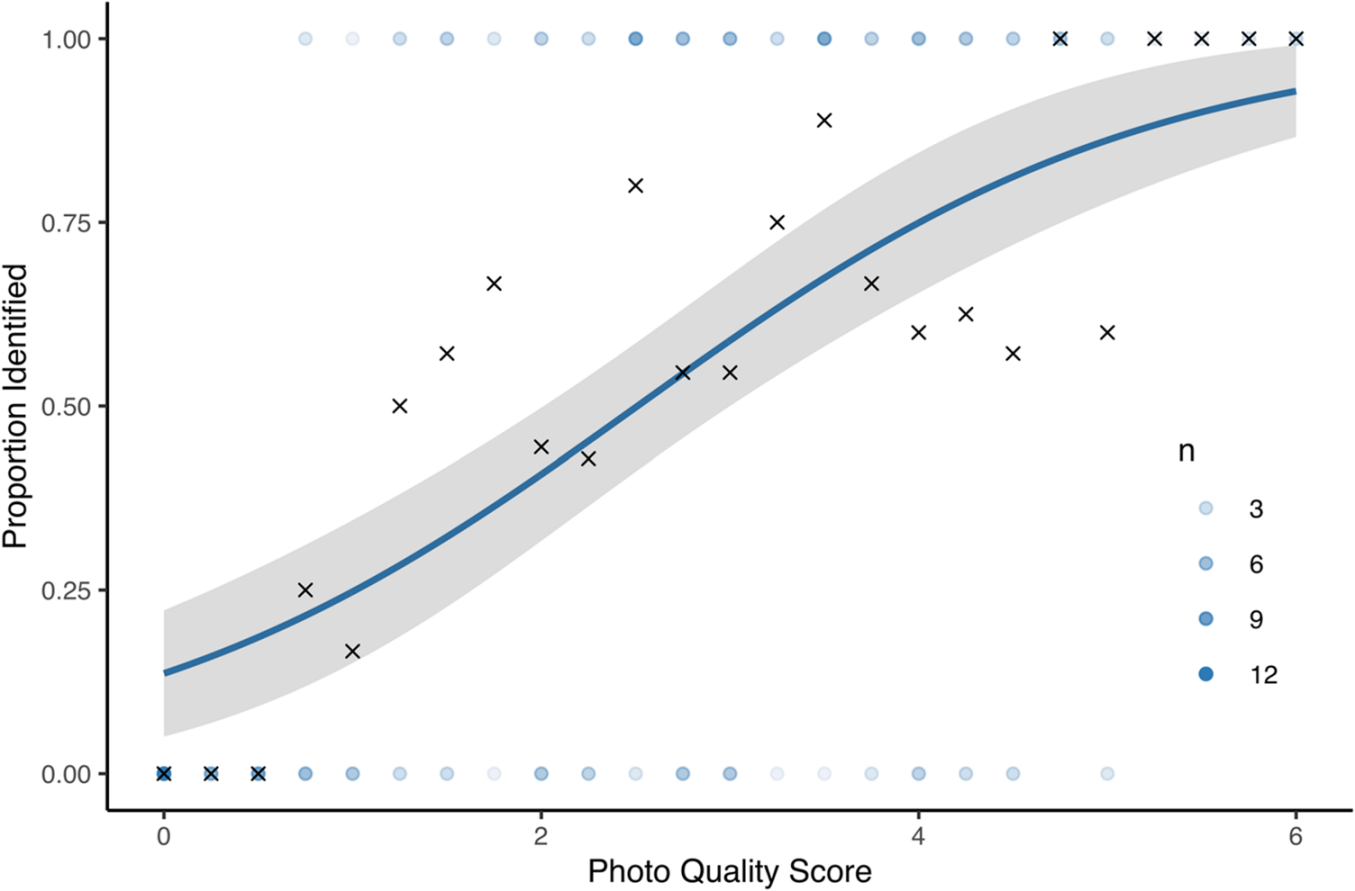
Predicted identifiability based on photo quality score (logistic regression, n = 168, McFaddens R-squared = 0.188). Shaded region is the 95% confidence interval. The sample size of bees at each photo quality score is represented by blue dots. X represents the proportion identified at each photo quality score.

A combined photo quality score (0–6) explained some of the variation in whether a species identification could be made from photographs (Fig. 6, McFadden’s R-squared = 0.188, *n* = 168). Of 168 bumblebees for which we scored photo quality, three had photo quality scores of six (all eight diagnostic features clearly visible in at least one photograph) and twelve had photo quality scores of zero (no key features clearly visible: Fig. 5). The average photo quality score was 3.33 (s.d. = 1.31, n = 84) for photos with a species identification and 1.76 (s.d. = 1.52, n = 82) for photos without a species identification. All bees with a score of over five had a species identification.

## Discussion

Our results show that for *Bombus* spp., photo identification with suitable photographs by trained experts can provide data of similar quality as identifications based on specimens, when comparing experts with substantial training. Species identifications of photographs generally had very high accuracy with 95.1% agreement with initial specimen identifications. Bumblebees are difficult to identify and identifications can differ across experts for both specimen and photo identifications based on multiple factors including experience and training in either technique. Our limited consideration of agreement through the lens of a consensus approach suggested that photo identification accuracy may be even higher than 95%; in cases where the specimen and photo identifications disagreed, the second specimen identification agreed almost as frequently with the photo identification as with the initial specimen identification. Therefore, experts using both methods likely misidentified a small percentage (2.5% for specimens, 2.7% for photographs) of bumblebees in the study. This approximately matches a study finding a single expert identifier had 95% consistency with themselves in identifying 16 bumblebee species in 100 photos^39^.

One approach to minimizing misidentifications involves gathering multiple opinions and aggregating the results. In one of the most thorough evaluations of photo identification success, a study of wildlife camera trap photos from the Zooniverse Serengeti project found that identifications made by an aggregation of citizen scientists agreed with a panel of experts on 97.9% of all images. This exceeded agreement of individual experts with the full panel, which averaged 96.6%^40^. Thus, having multiple people identify photographs, particularly for species known to be challenging to identify, may improve accuracy of identification. Machine-learning and similar artificial intelligence (AI) analysis techniques may also yield reasonable results for photographs of similar quality as those presented here if sufficient training data, illustrating the same characteristics that cue experts, exist and feedback on improving photo quality can be incorporated. These approaches may also serve as examples to train novice observers in correct identification with oversight from skilled taxonomists.

As illustrated above, small percentages of species misidentifications that can be common even among experts may have serious implications. False positives create problems for species of concern resulting in overestimation of population size or range and thus status of the population. Our dataset included a bee identified as *B. fervidus* from photographs, which is listed as vulnerable by the IUCN. However, both the initial and second expert evaluating the specimen identified the bee as a different species of concern, *B. pensylvanicus* (Table 1). Our data suggest that in Montana and the western edge of North and South Dakota, bees with more frequent misidentifications from both photos and specimens include *B. centralis, B. flavifrons, B. huntii* males, and *B. rufocinctus* (both sexes)*.* Specifically, *B. flavifrons* frequently was confused with *B. centralis*, and *B. rufocinctus, B. huntii, B. sylvicola,* and *B. frigidus* were frequently confused with each other (Fig. 3).

Bumblebees were less likely to be identified from photographs than from specimens (92 vs. 98%). If photographs do not capture key features and cannot be identified, and no specimen is collected, no opportunity exists to refer to the specimen to make an identification, resulting in a lost data point^37^. These unidentified bees can result in false negatives, not detecting a species when it is present. For example, in one instance we did not identify a *B. occidentalis* individual from photographs. Had we relied only on photo identifications, this could have resulted in a false negative or non-detection of *B. occidentalis* at this site. Interestingly, we also had one case where we were not able to identify a *B. occidentalis* individual from the specimen. Our participants had a range of experience in specimen preparation and this example serves as a reminder that while unidentifiable specimens were less common than unidentifiable photos neither method is perfect and that training is important to data quality. *Bombus occidentalis* is currently under consideration for listing under the Endangered Species Act^41,42^. Presence/absence data that are as free of false negatives as possible can support an accurate status assessment and effective management of this species.

Wildlife scientists and managers expect some false negatives in presence/absence research. Occupancy models account for false negative or non-detections and can thus provide more robust predictions for management^8^, but confirmed detection of a species in a location of interest provides certainty that can be useful for other kinds of analyses or decisions about where to implement conservation activities. Providing additional training for species of concern, at least for easily identified species, could maximize successful true positive detections. For this study, we trained technicians to identify *B. occidentalis* in the field to decrease false negatives for this species.

We found ensuring photographs have good focus, resolution, and lighting and include views of the key features needed to make identifications improved identifiability. Only three out of 168 sets of photographs for which we scored photo quality had clear views of all eight key features. Considering this, our results show how even imperfect photographs can be useful for identifications, while highlighting how important clear protocols and training are for reliable identifiability and accuracy. In addition to initial training, periodic quality assessment can help to identify improvements in technique.

We expected lower identification and agreement rates for male than female bumblebees. Identification of male bumblebees requires inspection of the genitalia, which makes males difficult to identify from photographs. We found males were slightly less likely to be identified overall, but the influence of sex on identification efficacy varied considerably by species. For some species, photographs of males were less likely to be identified, but we found no difference in misidentification. For example, *B. insularis* males and *B. nevadensis* males had similar rates of misidentification but were less likely to be identified from photographs compared to females of the same species. For other species, such as *B. huntii,* males were misidentified more frequently than females (Fig. 2). Finally, in some species such as *B. fervidus* and *B. griseocollis,* we found very little difference in identifiability or agreement between males and females. This suggests that while photo-based identification may not be an appropriate method for males of all species, in our study area it was highly effective for identification of *B. fervidus* and *B. griseocollis* males and may be for other easily identified species in other regions.

Beyond the influence of sex, our classification analysis identified three groups of characteristics that made photographs less likely to be identified. They were: (1) wet bees, (2) species with many color morphs and which did not include a clear view of the colors of the abdomen, and (3) photographs taken on an iPad, especially those without a good photo of the side of the thorax. Other identifying criteria with lower support included the visibility of the hind leg, face ‘hair’ color, and head ‘hair’ color, which depend on the image resolution. Minor adjustments to protocols likely can improve identification success even further. For instance, the color of ‘hairs’ is a key feature for identifying bumblebee species. Because we used ice to slow down bees for photographs, bumblebees got wet on hot days when condensation collected inside the vials and when water seeped in while in the cooler. When wet, ‘hairs’ become slicked back against bumblebee bodies and the color of the ‘hairs’ became difficult to distinguish. The top two alternative splits for the top-level classification were whether the colors of the abdominal ‘hairs’ and the colors of the thorax ‘hairs’ were clearly visible. For wet bees, clear views of these characteristic rarely occur. Inspecting vials and caps for cracks and ensuring vials are not submerged will increase the probability the bee stays dry. If bees do become wet, collecting the specimen and washing and drying the bee will increase the likelihood of an accurate identification. Similarly, we realized after our first year of sampling how much camera type mattered and provided crews previously using iPads with small digital cameras equipped with a focus stacking feature that created composite images for better depth of focus and a flash ring. These features resulted in photographs with more key features in focus and better preservation of color. We did not formally evaluate whether the extent that the bee filled the frame influenced identifiability as we could easily zoom in to better see any feature. We also did not consistently include a ruler that could help with identification of caste. Given that some problems may be specific to a particular approach used to obtain photos or idiosyncrasies of specific photographers, reviews of data quality early on can help to improve data quality.

Our finding that species with more color morphs were less likely to be identified through photographs, especially if a good photo of the abdomen was lacking, may be most difficult to address given that many species have multiple color morphs. In this speciose region, species with more color morphs have morphs that appear similar to other species, making it difficult to confidently distinguish species. We conducted this study in Montana and the western edge of the North and South Dakota (supplementary Fig. 2) in counties with 8 to 28 documented species, among the highest species richness in North America. The efficacy of photo identification likely varies by region, depending on the presence of difficult to distinguish and cryptic species as well as the number of species and color morphs. Lists of the bees and the color morphs of those species potentially present in the study area can inform strategies to both train data collectors and modify protocols. Solutions include providing additional training and requiring additional photos for similar bees. For example, in the Pacific Northwest Bumble Bee Atlas, scientists record an additional photograph of the underside of the abdomen for yellow-faced, yellow-striped bees^43^. Location information, habitat information, and specifics on the ranges of bee species and color morphs provide information valuable in our case that could be incorporated into artificial intelligence identifications, as long as incorporation of such information allows for the possibility of new species’ discoveries or range expansions.

Other influences on identifiability and correct identification could include training and experience in identification both overall and specific to the region, overconfidence in distinguishing difficult to identify species when photos provide limited detail, and the quality of location and habitat information provided with the photographs^44,45^. Here, one expert completed all photo identifications, and the results may not be representative of other experts. Finally, this study used an established sampling protocol with four to six photographs of bees that were chilled to slow their movement. Error rates are likely to differ with fewer photos per bee, more active bees, or of in situ bees (rather than those immobilized specifically for photo ID). In addition to training on identification of species of concern, recording the name of putative species of concern and relevant distinguishing features at capture will support extra attention for these cases. Taking extra care to ensure high quality photographs of those relevant features will likely improve data quality. For female bees in this study area, ensuring clear photos of the 4^th^ and 5th tergal segments will help identify *B. occidentalis*. Considering other species we were unable to accurately identify from photographs, taking clear photos of the color of the ‘hairs’ on the fourth tergal segment can separate female *B. fervidus* from *B. pensylvanicus* and photos that capture the extent and shape of black ‘hairs’ in the interalar band between the wings help distinguish *B. pensylvanicus* from *B. nevadensis*^46^.

Our analyses for this study matching photo identifications to specimens also led us to find several data entry errors, highlighting the importance of good protocols and data management to reduce such issues, whether using photo- or specimen-based identification. For instance, we found photographs of two different bees uploaded as part of one bee observation, photographs of the same bee uploaded multiple times, and photographs uploaded to the wrong survey location. Including relevant location and identifying information in each photograph and using the timestamp and location in the photo metadata helped in finding and correcting errors. Prompt and careful addition of photographs into databases likely reduces errors, just as occurs with other kinds of data entry. Similar issues can occur with specimens and careful pinning, curation, and labeling can improve data quality.

One other consideration for research and monitoring relates to other information gleaned from specimens. Creating synoptic collections of specimens for a sampling effort may balance objectives to minimize harm and maximize data and provide a reference collection for training novice participants and calibrating expert knowledge of an area. Specimens provide samples for uses such as genetics that have led to the realignment of species^47^, identification of cryptic speciation, and identification of pollen^48^. They have proven usefulness for other new technologies related to disease and isotopes and will likely form the basis for evaluation of distributions or other ecological questions in ways yet unimagined. Archiving photographs can similarly aid future training, research, and validation.

In a landscape of imperfect techniques for morphometric bumblebee identifications, we found photo identification performed comparably to specimen identifications with respect to the accuracy of identifications. Neither specimen nor photo identifications were 100% accurate, and, as a result, misidentification should be expected and accounted for through techniques such as multiple identifications or modeling. Although photo vouchers have limitations in comparison to a specimen in hand, using best practices outlined here can maximize identifiability based on photographs.

## Methods

### Data collection

We sampled bumblebees to answer multiple research and management questions in partnership with Bureau of Land Management Montana/Dakotas, U.S. Fish and Wildlife Service, and Glacier National Park^8,49^. Therefore, samples represent bumblebee populations from locations ranging from the eastern side of Glacier National Park to the very western edge of North Dakota and southwestern South Dakota.

At each sample site, observers completed timed hand net surveys for 45 person-minutes following protocols used in the Pacific Northwest Atlas project^50^. When a bee was caught, the timer was stopped to put the bee into a vial. Bees were chilled on ice for about 10 min to slow their movements and then were photographed. We took pictures of four key identification regions on each bee: abdomen, side body with hind legs, top of thorax, and face (Fig. 1). These four angles allowed us to efficiently capture key features for identification, including the characteristics of ‘hairs’ on the tibia of the hindleg, the cheek length, and the color of ‘hairs’ on tergal segments of the abdomen, the thorax, the face, and the top of the head^46,51^. For most sites, one specimen of each type that looked like a different species or caste from each survey was freeze-killed and collected. Cameras varied by survey and included: iPads, iPhones, Samsung tablets, and small digital cameras, including the Olympus TG5 which combines multiple images to create stacked macro images with good focus throughout the entire field of view.

### Identification

For all photographs, Rich Hatfield, who has been identifying bumblebees from specimens for more than twenty years and from photographs for over 10 years across multiple projects, determined the finest possible taxonomic level for bumblebees using Williams et al. (2014)^51^ for taxonomic concepts and descriptions, while also consulting species descriptions in Thorp et al. (1983) and Stephen (1957)^52,53^. Some bumblebees were only identified to genus. Amelia Dolan, who developed the key to Montana bumblebees through examination of ~ 12,000 specimens, provided initial determinations for all physical specimens using that key^46^. A second opinion on determinations of a subset of 23 specimens with species identifications from photographs that did not match specimen-based identifications, was provided by Casey Delphia, who has been identifying bumblebees and other wild bees in Montana for over 10 years and who is a coauthor on the key to Montana bumblebees^54,55^. All experts had access to the location bees were collected and knowledge of species and color morphs of species that could be in the region which informed identification.

For the first three years of the project, we collected photographs using the Survey123 app^56^ and photographs were reviewed in geodatabases stored on ArcGIS Pro^57^. In 2021, we uploaded photographs to Bumble Bee Watch (bumblebeewatch.org), which is optimized for photo-based identification of bees with features including a drop-down list of species.

### Data analysis

#### Measuring efficacy: identifiability and agreement

We measured the efficacy of bumblebee identification using two metrics: identifiability and agreement. We defined identifiability as the percentage of bumblebees for which experts identified the species from either photographs or specimens alone. We defined agreement as the percentage of species identifications from photographs that matched (agreed with) the species identification of the same bee made from the specimen. For the subset of bees with both specimens and photographs (n = 561), we also calculated species- and sex-specific identifiability and agreement scores. We used classification and regression analyses to evaluate how photo quality, bee characteristics, and camera type affected identifiability to inform best practices for improving identification rates.

To better understand whether disagreements between specimen and photo identifications resulted from a specimen misidentification or a photo misidentification, we used a consensus approach to compare the two methods. We defined the consensus identification as the species identification that was supported by at least two independent experts. In cases where the initial photo identification and specimen identification agreed on a species, this species was the consensus identification. In cases where the initial photo and specimen identifications disagreed and a second opinion specimen identification was available, the consensus identification was the species that was supported by at least two of the three independent identifications. We calculated the proportion of identifications that were supported by the consensus definition for both specimen and photo identifications.

#### Quantifying photo characteristics

Because describing photo quality required manual assessment of photographs, we created a representative subset of data for which to record these characteristics. To create the subset, we randomly sampled a subset of identified bees for comparison with unidentified bees, stratifying by species and sex.

We scored photo quality based on whether eight key features were clearly visible in at least one photograph of each bee. The first feature was the corbicula or other hairs on the hind leg and the other seven features were colors of the ‘hairs’ on the: tergal segments (on the abdomen), the posterior two segments of the abdomen, the abdomen under the wings (seen from above), the thorax between the wings, the side of the thorax, the face, and on the top of the head. We selected these features because they were (1) important for distinguishing between species in this region^46,51^ and (2) the individual scoring photo quality could consistently decide if the photo included the feature in sufficient detail to be useful for identification. The tibia of the hindleg is used to distinguish between true bumblebees and cuckoo bumblebees^51^. Although important for identification, we excluded cheek length because it was difficult to consistently score. For each feature, we gave a bee a score of 1 if the feature was clearly visible or a 0 if it was not clearly visible. In intermediate cases, e.g., we could see yellow ‘hairs’ in the face area of a photo, but there was not sufficient resolution or focus to see if black ‘hairs’ were mixed in, we gave the feature a score of 0.5. We also scored photos for whether the bumblebee subject was wet or matted using the same 0 to 1 scale. All scoring was done by one individual (Colgan).

#### Characteristics influencing identification

We used classification tree analysis to identify factors that were predictive of whether an identification could be made from photographs. Because we had < 10% of bees that were not identified from photographs and many statistical frameworks behave poorly on the boundaries, we created a stratified random sample for this assessment. We selected equal numbers of bees identified in photographs as those not identified from photographs in each sex/species group. Classification tree approaches are robust to unequal variance, allow missing values in predictor variables, and can include both numeric and categorical predictor variables^58^. In addition to the nine photo quality characteristics described above and the type of camera used to take the photographs, we considered whether the bee was a cuckoo bumblebee, had white on the body, had red on the body, had a black tail, or was striped. (supplementary Table 3). Color classifications reflected categories described in the guide to Pacific Northwest female bumblebee species from the Pacific Northwest Bumble Bee Atlas^50^. Cuckoo bumblebees are parasitic and can be differentiated from true bumblebees because they lack a corbicula for collecting pollen^50^. We conducted the classification analysis using the package mvpart^59^. When used with a univariate response variable, as here, the mvpart function specifying the method, class, calls the rpart function from the rpart package, which is an implementation of classification and regression tree analysis that closely follows the method described by Breiman et al.^60,61^. The approach identifies the variable that best splits the data into two groups at each successive node based on minimizing the Gini index. We selected the pruned tree with the smallest cross-validated relative error based on 100 cross-validations.

To better understand the relationship between photo quality and identifiability, we fit a logistic regression relating overall photo quality score to identifiability, a binary response variable, using the glm function from the R stats package^62^. To calculate the overall photo quality score, we created a weighted sum of the eight key features as follows: the four primary key features (abdomen, thorax, head, and face) were given a weight of 1 and the four secondary features (abdomen under wings, end of the abdomen, side thorax, and hind leg) were given a weight of 0.5. Thus, the highest possible score was 6 and the lowest possible score was 0. We evaluated goodness of fit for the logistic regression using McFadden’s R-squared^63^. We conducted all analysis in the statistical software environment R version 4.1.1^62^.

### Supplementary Information

Supplementary Information.

## Data Availability

The datasets generated and analyzed during the current study are available in Graves (2022)^64^ at https://doi.org/10.5066/P931YWY8 Photographs and specimens can be reviewed through contact with the corresponding author on reasonable request.
