# Supplementary material for: Quantifying effectiveness and best practices for bumblebee identification from photographs
Source: Sci Rep. 2024 Jan 10;14:830. doi: 10.1038/s41598-023-41548-w (PMC10782012; doi:10.1038/s41598-023-41548-w)
Supplement: Supplementary file 1 — Supplementary Information. [file 41598_2023_41548_MOESM1_ESM.pdf]

Supplement for:
Quantifying Effectiveness and Best Practices for Bumblebee Identification from Photographs
Scientific Reports, https://doi.org/10.1038/s41598-023-41548-w

A. M. Colgan[1], R. G. Hatfield[2], A. Dolan[3], W. Velman[4], R. E. Newton[4], T. A. Graves[5]

[1] Contractor to the U.S. Geological Survey, West Glacier, MT 59936, USA
[2] Xerces Society for Invertebrate Conservation, 628 NE Broadway, Suite 200, Portland, OR 97221, USA
[3] Boise, ID 83702, USA
[4] Bureau of Land Management, 5001 Southgate Drive, Billings, MT 59101, USA
[5] U.S. Geological Survey, Northern Rocky Mountain Science Center, 38 Mather Drive, PO Box 169, West Glacier, MT 59936, USA

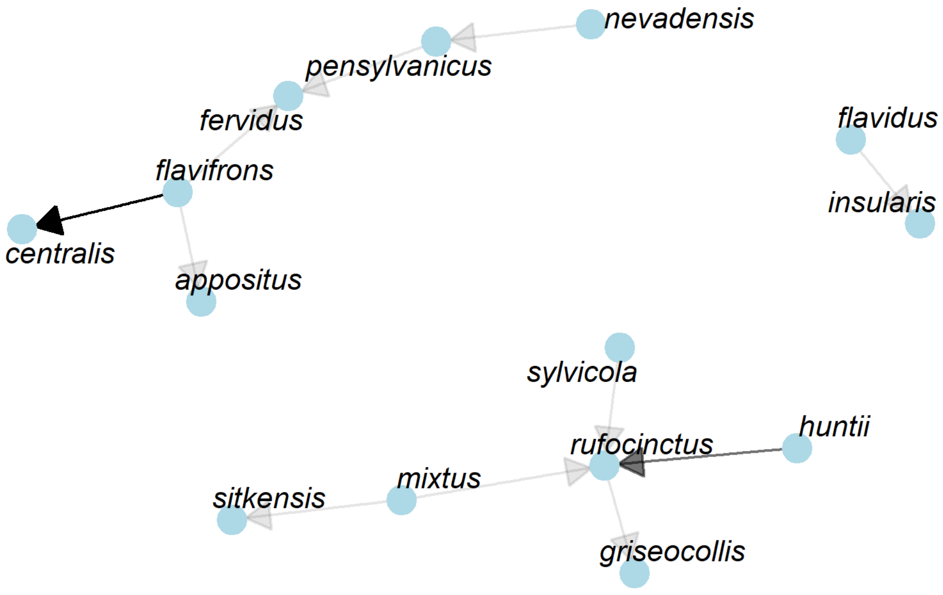

Supplementary Figure 1. Network diagram showing species confusions for bees where both initial and second opinion specimen determinations were different than the photo determination. Arrows point from the specimen identification to the photo identification. Darker arrows indicate more frequent confusion between two species.

Supplementary Table 1. Efficacy of bumble bee identification from photographs reported by species relative to specimen identifications for 551 bees collected 2019-2021 in Montana, North Dakota, or South Dakota, USA. Percent photos identified is the percent of bees for which a species level identification was able to be made from photographs. Percent agreement is the percent of bees for which the photo identification matched the specimen identification. In cases where the photo identification did not match the specimen identification, we report the photo identification in the corresponding "confused with" column. Blue values indicate better than average scores, red values indicate worse than average scores, greyed out values indicate <10 samples. Photo identifications are reported in cases where they disagreed with specimen identifications and are bolded when supported by a second opinion specimen identification.

**Specimen ID**	**% Photos Identified (*n*)**	**% Agreement (*n*)**	**Photo ID (*n*)**	**Second Opinion Agreed With (*n*)**
B. appositus	80 (5)	100 (4)		
B. bifarius	92.6 (54)	100 (50)		
B. bimaculatus	50 (2)	100 (1)		
B. centralis	75 (20)	93.3 (15)	***B. huntii (1)***	*Photo (1)*
B. fervidus	94.1 (68)	100 (64)		
B. flavidus	88.9 (9)	87.5 (8)	*B. insularis (1)*	*Specimen (1)*
B. flavifrons	76.2 (21)	75.0 (16)	*B. appositus (1), B. centralis (3)*	*Specimens (4)*
B. fraternus	100 (1)	100 (1)		
B. griseocollis	96.8 (93)	98.9 (90)	*B. rufocinctus (1)*	*Photo (1)*
B. huntii	80.8 (78)	92.1 (63)	***B. bifarius (1),*** *B. rufocinctus (2),* ***B. sylvicola (2)***	*Specimen (1), Photos (3), neither (1)*
B. impatiens	100 (1)	100 (1)		
B. insularis	76.7 (43)	100 (33)		
B. melanopygus	100 (3)	100 (3)		
B. mixtus	72.7 (11)	75 (8)	*B. rufocinctus (1), B. sitkensis (1)*	*Specimens (2)*
B. nevadensis	90.5 (63)	98.2 (57)	*B. pensylvanicus (1)*	*Specimen (1)*
B. occidentalis	50 (2)	100 (1)		
B. pensylvanicus	100 (7)	85.7 (7)	*B. fervidus (1)*	*Neither (1)*
B. rufocinctus	88.9 (54)	87.5 (48)	***B. frigidus (3),*** *B. griseocollis (1),* ***B. huntii (1), B. sylvicola (1)***	*Specimen (1), Photos (5)*
B. sylvicola	100 (6)	66.7 (6)	*B. rufocinctus (2)*	*Specimen (1)*
B. vagans	100 (10)	100 (10)		

Supplementary Table 2. Efficacy of specimen bumble bee determination relative to identifications from photos for 551 bees collected 2019-2021 in Montana, North Dakota, or South Dakota, USA. Species is based on photograph species determination. Percent specimens identified is the percent of bees for which an expert determined a species level identification. Percent agreement is the percent of bees for which the specimen identification matched the photo identification.

**Photo Species Determination**	**Sample Size**	**% Specimens Identified**	**% Specimen Agreement with Photograph**	**Confused with**
B. appositus	5	100	80	*B. flavifrons*
B. bifarius	51	100	98	*B. huntii*
B. bimaculatus	1	100	100	
B. centralis	20	85	82.4	*B. flavifrons*
B. fervidus	65	100	96.9	*B. flavifrons, B. pensylvanicus*
B. flavidus	7	100	100	
B. flavifrons	12	100	100	
B. fraternus	1	100	100	
B. frigidus	3	100	0	*B. rufocinctus*
B. griseocollis	90	100	98.9	*B. rufocinctus*
B. huntii	60	100	96.7	*B. centralis, B. rufocinctus*
B. impatiens	1	100	100	
B. insularis	34	100	97.1	*B. flavidus*
B. melanopygus	3	100	100	
B. mixtus	7	85.7	100	
B. nevadensis	57	98.2	98.2	*B. fervidus*
B. occidentalis	2	50	100	
B. pensylvanicus	7	100	85.7	*B. nevadensis*
B. rufocinctus	48	100	87.5	*B. griseocollis, B. huntii, B. mixtus, B. sylvicola*
B. sitkensis	1	100	0	*B. mixtus*
B. sylvicola	7	100	57.1	*B. huntii, B. rufocinctus*
B. vagans	10	100	100	

Supplementary Table 3. Properties of bee species. Color categories are based on "Pacific Northwest Identification Guide for Female Bumble Bees" from the Pacific Northwest Bumble Bee Atlas. *Bombus pensylvanicus* is an eastern species that was added. Number of color morphs based on USDA Forest Service's "Bumble Bees of the Western United States" and "Bumble Bees of the Eastern United Stated". Where a species was included in the western guide, the number of color morphs reported in that guide was used (western). If not, the number of color morphs reported in the eastern guide was used (eastern). Blanks indicate this information was not available for the species in either guide.

**Bombus Species**	**IUCN Status**	**Striped Bumble Bees**	**Red on Body Bumble Bees**	**Black Tailed Bumble Bees**	**White on Body bumble Bees**	**Number of Color Morphs**	**Source**
B. appositus	LC	0	0	0	1	12	western
B. bifarius	LC	1	1	0	0	12	western
B. bimaculatus	LC					16	eastern
B. centralis	LC	0	1	0	0	5	western
B. fervidus	VU	1	0	1	0	9	western
B. flavidus	LC	0	0	0	0		
B. flavifrons	LC	0	1	1	0	15	western
B. fraternus	EN					6	eastern
B. griseocollis	LC	0	0	1	0	17	western
B. huntii	LC	0	1	0	0	5	western
B. impatiens	LC					4	eastern
B. insularis	LC	0	0	0	0	9	western
B. melanopygus	LC	1	1	0	0	13	western
B. mixtus	LC	0	1	0	0	9	western
B. nevadensis	LC	0	0	1	0	8	western
B. occidentalis	VU	0	0	0	1	12	western
B. pensylvanicus	VU	0	0	1	0	13	eastern
B. rufocinctus	LC	1	1	0	0	21	western
B. sylvicola	LC	1	1	0	0	14	western
B. vagans	LC	0	0	1	0	9	western

Supplementary Table 4. Summary of sample used in classification analysis. Using the specimen identification, we selected an equal number of bees identified in photographs (84) as those unidentified (84) for each sex/species group as much as possible. This balanced sample size removed a challenge in classification analyses when classes (identified/unidentified) are extremely unbalanced, having one class occur as <10% of the total and created a dataset with a reasonable sample size to record all characteristics and have meaningful results.

**Specimen ID**	**sex**	***n* Identified**	***n* Unidentified**
B. appositus	female	1	1
B. bifarius	female	4	4
B. bimaculatus	male	0	1
B. centralis	female	2	2
B. centralis	male	3	3
B. fervidus	female	3	2
B. fervidus	male	1	1
B. flavidus	female	1	0
B. flavidus	male	1	1
B. flavifrons	female	4	5
B. griseocollis	female	1	1
B. griseocollis	male	2	2
B. huntii	female	10	10
B. huntii	male	4	4
B. insularis	female	1	1
B. insularis	male	9	9
B. mixtus	female	2	2
B. mixtus	unknown	0	1
B. nevadensis	male	6	6
B. occidentalis	female	1	1
B. rufocinctus	female	4	3
B. rufocinctus	male	2	2
sp.	female	3	3
sp.	unknown	0	1
No specimen	female	9	6
No specimen	male	5	4
No Specimen	unknown	5	8
Total		84	84

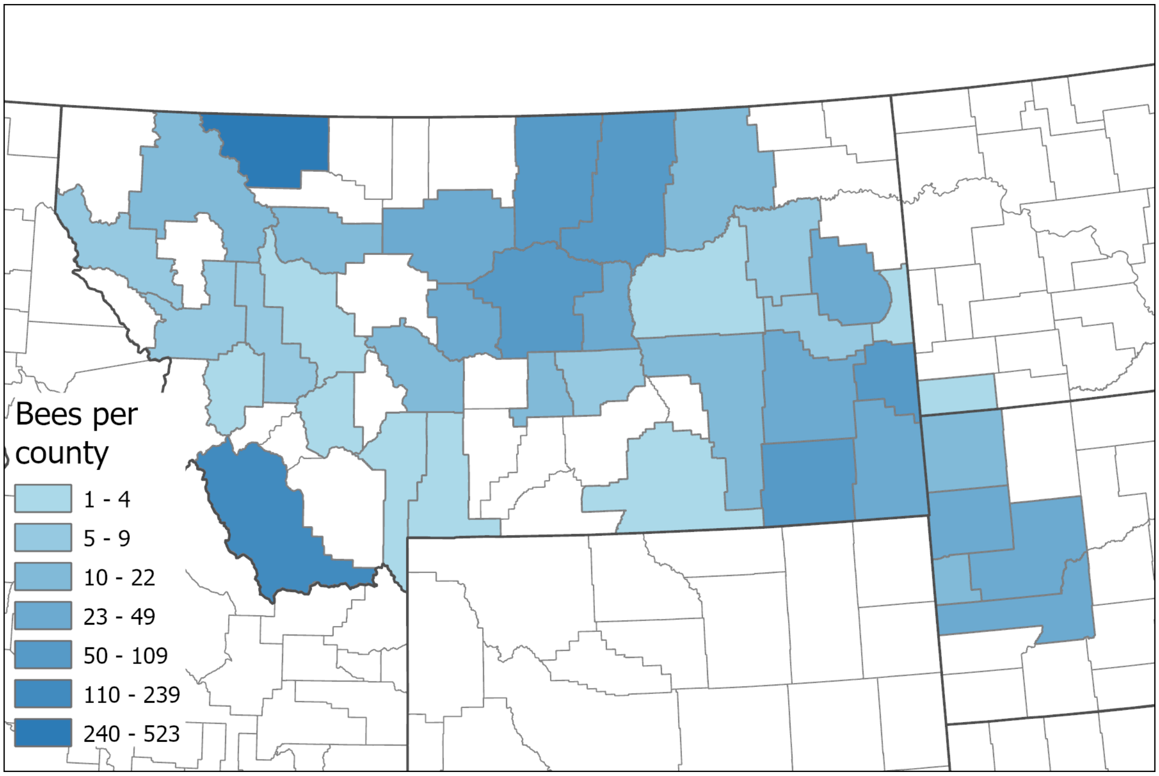

Supplementary Figure 2. Spatial distribution of collection locations for bees included in the analysis. We generated this map in ArcGIS Pro [GIS software], version 2.9, https://pro.arcgis.com/en/pro-app/2.9/get-started/download-arcgis-pro.htm using data published in Graves et al. (2022). Other data on bees in Montana is available through the Montana Natural Heritage Program.

Any use of trade, firm, or product names is for descriptive purposes only and does not imply endorsement by the U.S. Government.

Graves, T.A., 2022, Bumblebee Surveys in Montana, North Dakota, South Dakota, and Nevada, USA (ver. 2.0, April 2023): U.S. Geological Survey data release, https://doi.org/10.5066/P931YWY8.
